# Supplementary material for: Astrocyte- and Neuron-Derived Extracellular Vesicles from Alzheimer’s Disease Patients Effect Complement-Mediated Neurotoxicity
Source: Cells. 2020 Jul 4;9(7):1618. doi: 10.3390/cells9071618 (PMC7407141; doi:10.3390/cells9071618)
Supplement: Supplementary file 1 [file cells-09-01618-s001.pdf]

Supplementary Table 1. Subjects demographics and clinical parameters. 5. Supplementary information.

	**AD**	**Normal**	**FTLD**	***p*-Value (AD vs. Controls/ AD vs. FTLD)**
Number of subjects	7	6	2	---
Age (years)	73 ± 9	76 ± 7	78 ± 21	0.545/ 0.589
Sex	5F/2M	4F/2M	1F/1M	
MMSE	22 ± 5	---	23 ± 9	--- / 0.805
ADAS-cog	18 ± 5		20 ± 21	--- / 0.744
CDR global	0.6 ± 0.2	---	0.7 ± 0.3	--- / 0.345
CDR-sob	3.2 ± 2	---	3.5 ± 3	--- / 0.880
CSF Total Tau	61 ± 12	---	58 ± 33	--- / 0.848
CSF p181-Tau	43 ± 13	---	31 ± 13	--- / 0.283
CSF $A\beta_{1\text{-}42}$	168 ± 28	---	237 ± 3	--- / 0.014

Variables except for sex are presented as mean ± standard deviation. P-values for differences were calculated using unpaired t-test. M = males; F = females. MMSE, Mini-Mental State Exam score; ADAS-cog, Alzheimer's Disease Assessment Scale-cognitive subscale score; CDR, Clinical Dementia Rating scale. Plasma samples of 13 additional individuals from the Johns Hopkins Alzheimer's Disease Center (JH ADC) with autopsy-confirmed AD were used in this study. However, these are not included in the table given that limited information on cognitive performance and other variables was provided.

1. Supplementary Methods

Assessment of CD59 protein levels in EVs by ELISA. The abundance of the endogenous complement inhibitor CD59 in undiluted MPER-lysed AEVs, NEVs and CD81+ EVs of AD patients and normal controls was evaluated using ELISA (cat. no. ELH-CD59, RayBiotech, Norcross, GA). The protein levels of CD81 were used as a normalization factor for EV load (cat. no. CSB-EL004960HU, Cusabio Technology, College Park, MD). ELISA plates were read using the multifunctional microplate reader Synergy™ H1 and data collected using the Gen5™ microplate data collection and analysis software (BioTek Instruments, Winooski, VT). Absorbance signals above the limit of detection (LoD) were not extrapolated to protein concentration as they were below the lowest limit of quantification (LLoQ) and are reported as raw signal. The LoD was defined as the mean signal of the diluent blank plus 2.5 times its standard deviation whereas the LLoQ was determined by the standard solution with signal above the LoD, CV among duplicates lower than 20%, and recovery between 80% and 120%.

2. Assessment of EV Uptake by Neurons

To evaluate the interaction of AEVs with recipient neurons, E18 rat cortical neurons at 21 DIV were treated with fluorescently labelled AEVs which were then tracked using fluorescence confocal microscopy to elucidate their sub-cellular localization. AEVs isolated from AD and normal participants were labelled with the red fluorescent lipid analogue PKH26 (Sigma Aldrich, St. Louis, MO) as instructed by the manufacturer with modifications. 100 µL of AEVs at a concentration of 10^{10} EVs/mL (10^9 particles) by NTA were incubated with 5 µL of a 4 µM PKH26 solution in diluent C for 1 min at RT (diluent C lacks physiological salts and thus reduces the formation of dye micelles that could result in a false positive neuronal staining) [90]. To control for the possibility that salts in the EV vehicle, i.e. DPBS, could induce the formation of dye micelles and thus interfere with the interpretation of results, we subjected DPBS alone to PKH26 using the same staining procedure as AEVs. To further minimize the potential for micelle

formation, after EV labelling, 400 μL of DPBS were added to the solution followed by the removal of free dye using size-exclusion chromatography (Zeba™ spin desalting column; cat. no. 89892, Thermo Fisher Scientific). The concentration of PKH26+ AEVs was assessed by NTA and 300 μL were added to E18 rat cortical neurons cultured for 21 DIV in PEI-coated wells of a borosilicate 8-well chambered coverglass (Thermo Fisher Scientific) at a concentration of 200,000 neurons per well in a final volume of 500 μL of neurobasal media (EV concentration in culture: 3-8 x 10^9 EVs/mL). Neurons were incubated with PKH26+ AEVs for 1 hour, washed two times with DPBS and fixed with 4% formaldehyde (FA) in DPBS for 10 minutes at RT. Excess FA was washed three times with DPBS. ProLong™ Diamond Antifade Mountant with the nucleic acid stain DAPI (Thermo Fisher Scientific) was added prior to visualization of cells using an LSM 710 confocal laser scanning microscope (Zeiss).

3. Trypsination of Extracellular Vesicles.

External EV protein epitopes were proteolytically cleaved using trypsin in order to evaluate whether neuronal uptake and EV-mediated neurotoxicity depend upon surface protein moieties. Trypsination of EVs was carried out using trypsin immobilized on beaded agarose (immobilized TPCK trypsin; Thermo Scientific, Inc.) to eliminate trypsin carryover to subsequent neuronal treatments and to avoid it causing EV permeabilization. 30 μL of immunoprecipitated EV samples ranging from 1-10 x 10^{10} EVs/mL (0.5-1 mg/mL total protein) were incubated with 10 μL of washed trypsin bead slurry (recommended usage based on trypsin activity: 200 μL trypsin bead slurry per mg of total protein) for 30 minutes at 37°C. Then, the trypsin gel was separated from the digestion mixture by centrifugation and trypsinized EVs in the supernatant were transferred to sterile microtubes. EV concentration and structure was assessed prior to neuronal treatments using NTA.

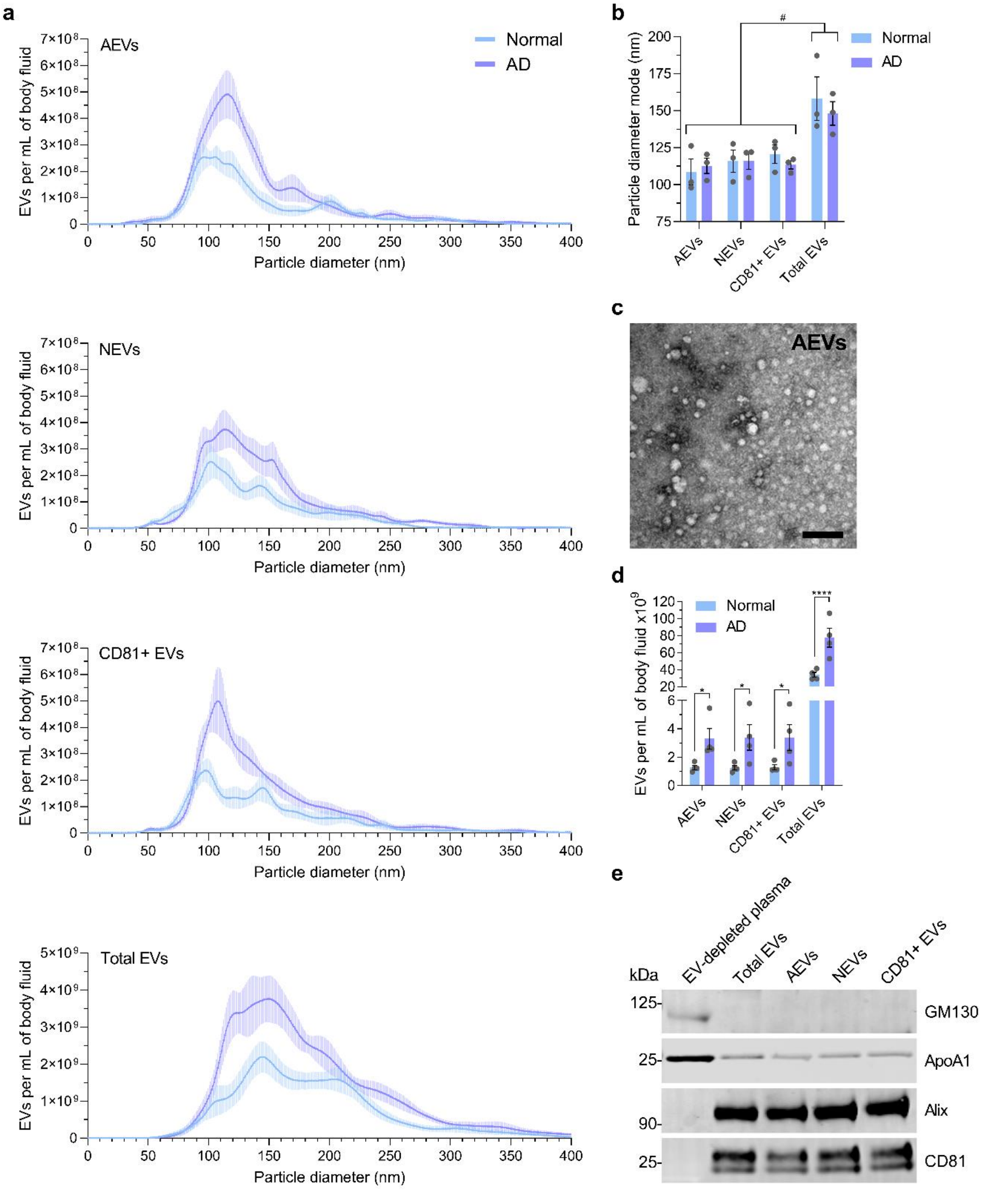

Supplementary figure 1. Characterization of extracellular vesicles. (**a**) The graph presents EV concentration (particles/EVs per mL of plasma) as a function of particle diameter [determined using nanoparticle tracking analysis (NTA)] for immunoprecipitated AEVs, NEVs and CD81+ EVs, and total EVs isolated from the plasma of 3 AD patients and 3 normal controls. (**b**) A bar graph showing the mean ± SEM of the particle diameter mode in **a** with individual values in grey. Statistical analysis: two-way ANOVA; significant differences indicated by the symbol '#'. (**c**) Transmission Electron Microscopy negative staining of AEVs of a normal aging participant. Scale bar, 200 nm. (**d**) A bar graph showing the mean ± SEM of the

particle concentration in **a** with individual values in grey. Statistical analysis: two-way ANOVA of AD vs Normal; AEVs: $*p = 0.0214$, NEVs: $*p = 0.0152$, CD81+ EVs: $*p = 0.0175$, total EVs: $****p < 0.0001$. Interestingly, all types of EVs from AD patients had higher particle concentrations compared to EVs from normal controls. **(e)** Western blots showing the protein levels of Apolipoprotein A1 as an indicator of lipoproteins co-precipitated with EVs, the cis-Golgi marker GM-130 used as a negative EV marker, and the transmembrane and intra-vesicular positive EV markers, CD81 and alix respectively, in 1 μg of total protein from the EV-depleted plasma, total EVs, AEVs, NEVs and CD81+ EVs of a normal control participant.

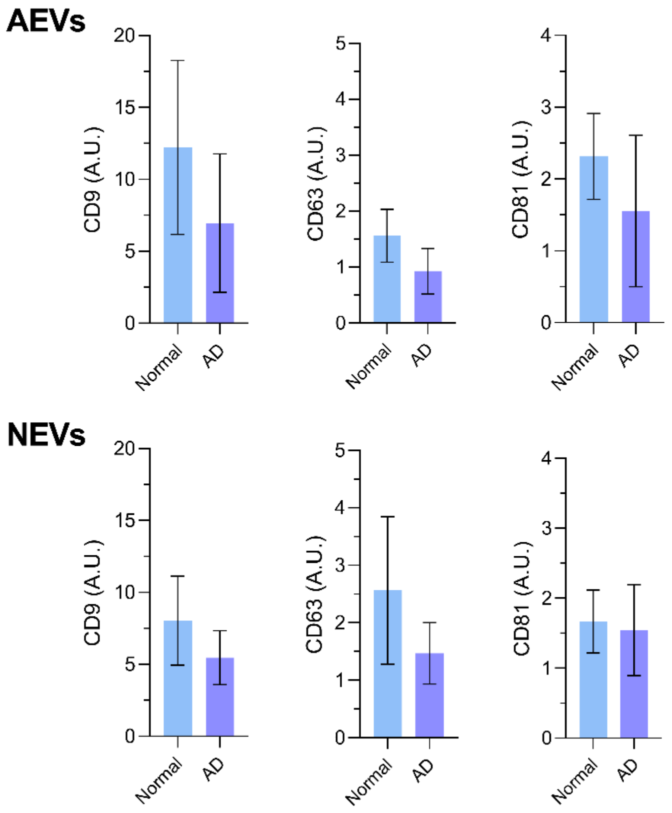

Supplementary figure 2. Levels of common membrane EV markers in intact AD AEVs and NEVs are similar to normal controls. Bar graphs showing the mean ± SD of the chemiluminescent signal corresponding to the protein levels of transmembrane EV markers CD9, CD63 and CD81 in intact AEVs (top) and NEVs (bottom) immunoprecipitated from the plasma of AD (n = 13) and control (n = 3) JH ADC participants. No significant differences were found between groups for any of the analytes assessed (statistical analysis: two-tailed unpaired t-tests). These results further suggest that the neurotoxicity of circulating AEVs from AD subjects cannot be attributed to differences in general EV markers or EV concentration.

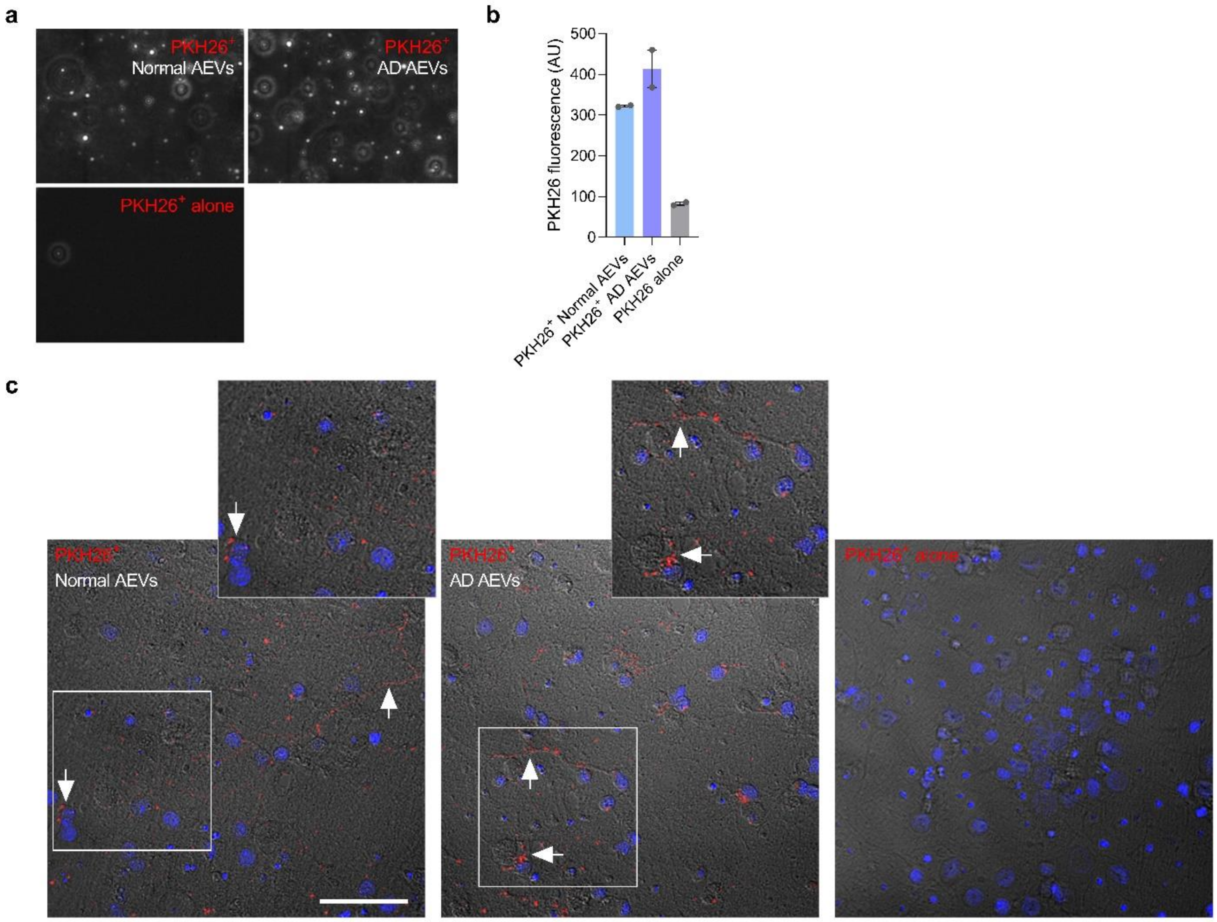

Supplementary figure 3. Neuronal internalization of AEVs. (**a**) Nanoparticle tracking analysis video stills showing that the particles collected by size exclusion chromatography after labelling with PKH26 (PKH26+ AEVs from normal control and AD participants) are not PKH26 micelles (PKH26 alone). (**b**) Mean PKH26 fluorescence intensity ± SEM of labelled AEVs from 2 AD patients and 2 normal controls compared to PKH26 alone confirm EV labelling. (**c**) PKH26 (red) fluorescence microscopy images merged with its differential interface contrast (DIC) and DAPI nuclear counterstain (blue) of rat cortical neurons treated with PKH26+ AEVs from normal control and AD participants for 1 hour show the accumulation of AD and normal AEVs in the soma and neurites (arrows in image and magnified inserts). Treatment with PKH26 alone was used as a negative control. Scale bar, 200 μm.

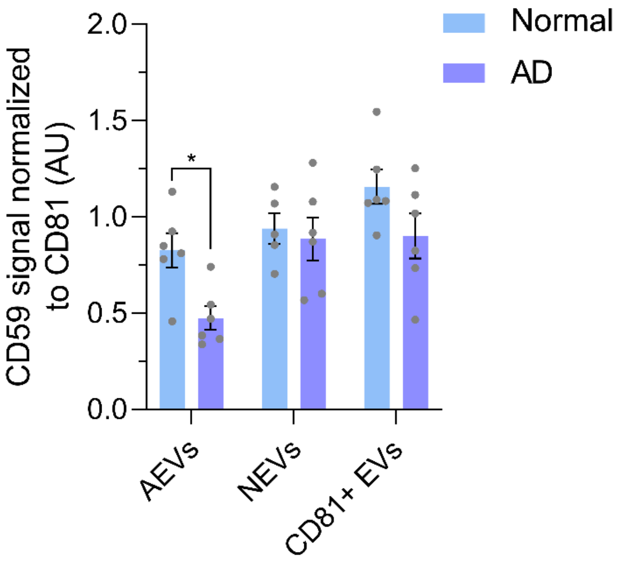

Supplementary figure 4. Decreased protein levels of the endogenous MAC inhibitor CD59 in AD AEVs. The protein levels of the endogenous MAC inhibitor CD59 was assessed in lysates of plasma AEVs, NEVs and CD81+ EVs of AD and normal control participants using ELISA. Each bar represents the mean value ± SEM of CD59 absorbance normalized to the EV input measured by CD81 ELISA from EV lysates of 6 AD subjects and 6 controls. Statistical analyses: two-way ANOVA of AD vs Normal; *p = 0.0360.

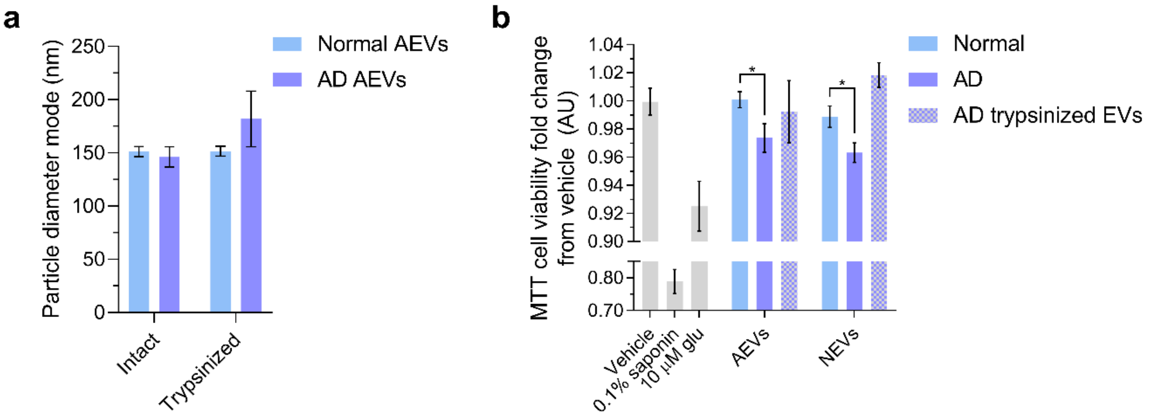

Supplementary figure 5. Trypsination of extracellular vesicles abolishes the neurotoxicity of AD AEVs and NEVs. (**a**) A bar graph showing that the mean NTA particle diameter mode ± SEM of intact AEVs of 2 AD and 2 normal control participants does not change after trypsination, thus suggesting that digestion of surface tryptic peptides does not disrupt EVs. (**b**) Bar graph showing that trypsination of AD AEVs and NEVs abolishes the significant decrease in MTT cell viability (fold change from vehicle) in E18 rat cortical neurons treated with AEVs and NEVs from 2 AD participants compared to neurons treated with AEVs and NEVs from 2 normal controls, respectively. Neurons treated with 0.1% saponin detergent and 10 μM glutamate were used as positive controls for neurotoxicity. Each bar represents the mean value ± SEM from triplicate wells and two different experiments. Analysis was based on multiple comparisons using one-way ANOVA: AD vs Normal AEVs, *p = 0.0238; AD vs Normal NEVs, *p = 0.0247.
